# Development and Characterization of Inkjet Printed Edible Films for Buccal Delivery of B-Complex Vitamins

**DOI:** 10.3390/ph13090203

**Published:** 2020-08-20

**Authors:** Georgios Eleftheriadis, Paraskevi Kyriaki Monou, Eleftherios Andriotis, Elisavet Mitsouli, Nikoleta Moutafidou, Catherine Markopoulou, Nikolaos Bouropoulos, Dimitrios Fatouros

**Affiliations:** 1 Laboratory of Pharmaceutical Technology, School of Pharmacy, Aristotle University of Thessaloniki, 54124 Thessaloniki, Greece; paraskemd@pharm.auth.gr (P.K.M.); andrioti@pharm.auth.gr (E.A.); mitsoulielsa@gmail.com (E.M.); dfatouro@pharm.auth.gr (D.F.); 2 Laboratory of Pharmaceutical Analysis, School of Pharmacy, Aristotle University of Thessaloniki, 54124 Thessaloniki, Greece; nikoletam@pharm.auth.gr (N.M.); amarkopo@pharm.auth.gr (C.M.); 3 Department of Materials Science, University of Patras, Rio, 26504 Patras, Greece; 4 Foundation for Research and Technology Hellas, Institute of Chemical Engineering and High Temperature, Chemical Processes, 26504 Patras, Greece

**Keywords:** buccal delivery, inkjet printing, B-complex vitamins, thiamine hydrochloride, nicotinic acid, release

## Abstract

Buccal films containing two vitamins, i.e., thiamine hydrochloride (THCl) and nicotinic acid (NA), were fabricated via two-dimensional (2D) inkjet printing. For the preparation of buccal films, solubility studies and rheological evaluations were conducted in distilled water and propylene-glycol (PG) as main solvent and viscosity/surface tension modifier, respectively. The increased solubility in the solvents’ mixture indicated that manufacturing of several doses of the THCl and NA is achievable. Various doses were deposited onto sugar-sheet substrates, by increasing the number of printing passes. The physiochemical characterization (SEM, DSC, FTIR) revealed that inkjet printing does not affect the solid state of the matrix. Water uptake studies were conducted, to compare the different vitamin-loaded formulations. The in vitro release studies indicated the burst release of both vitamins within 10 min, a preferable feature for buccal administration. The in vitro permeation studies indicated that higher concentrations of the vitamins onto the sugar sheet improved the in vitro permeation performance of printed formulations.

## 1. Introduction

In the last decade, printing technologies have gained popularity in the pharmaceutical field. Inkjet printing, in particular, is a non-contact method for alternative manufacturing of orally delivered formulations [1]. Drop-on-demand thermal inkjet printing provides a number of advantages in pharmaceutical technology. It facilitates the deposition of small amounts of liquids onto edible substrates, while the formulation is based on predesigned digital patterns [2]. The procedure is based on the generation of thermal pulses and the subsequent formation of a vapor bubble which expands and forces the liquid ink through the nozzle of the printer’s cartridge. Thus, a liquid droplet is formed directly and sprayed onto the substrate [3].

Personalized dosing is a new aspect in the pharmaceutical field and printing technologies are promising formulation approaches toward this direction, as they can produce various doses of one’s medicine in a tailored way [4]. Age, gender, and other genomic features vary among individuals, and as a result various doses of a medicine should be available for each patient in a fast and safe way [5]. Furthermore, the 2D inkjet printing technology provides the potential to advance the loading of poorly soluble drugs in a medicinal formulation, following the exploitation of suitable solvents in the development of inks [6]. The inkjet printing technique has been coupled with other formulation approaches, to fabricate hybrid drug delivery systems [7,8,9].

In order to achieve the optimal printability of the ink, the rheology of the liquid is the main factor that should be investigated. Specifically, viscosity and surface tension are critical parameters, as viscous liquids advance the formation of clogs and low-viscosity liquids would flow freely through the nozzle of the cartridge [10,11]. The use of viscosity and surface tension modifiers is imperative to produce a printable ink. Surfactants and polyhydric alcohols have been reported to effectively modify the rheological properties of the ink and to improve the solubility of several drugs.

Prednisolone and folic acid are representative active pharmaceutical compounds (APIs) that exhibit low solubility in the most common solvents; however these compounds were successfully formulated into oral dosage forms by inkjet printing [12,13]. Loperamide and caffeine were used as model drugs in order to investigate the preparation of flexible doses by inkjet printing [14], whereas the utilization of different substrates for loading rasagiline mesylate has been reported [15] Paracetamol, theophylline and caffeine were printed in porous substrates, presenting an alternative approach to control the deposition and the crystallization of the drugs [16]. Levothyroxine has been formulated by inkjet printing in order to develop flexible pediatric dosage forms [17]. Combining inkjet printing with the Quick Response (QR) technology also seems very promising, as practical information is encoded in drug loaded QR patterns (expiration date, route of administration, batch number, and manufacturer ID) and smart dosage forms are being formed for every individual [18,19].

The buccal delivery of APIs is an alternative, non-invasive route of administration that provides beneficial health effects. The buccal mucosa is a highly vascularized administration site, providing the high permeability for many APIs and the avoidance of the first pass effect [20]. Buccal formulations are recommended for pediatric dosage forms as they are patient-friendly and adjustable to the different physiological and pharmacokinetic profiles of infants, children and adolescents [21]. Clobetasol, omeprazole, and hydrocortisone are representative model drugs which have been formulated for buccal delivery [22,23,24]. The applicability of various printing technologies for patient-centric buccal formulations has been demonstrated by previous reports, in terms of (i) incorporating personalized doses or thermolabile drugs, and (ii) fabricating multi-layered buccal films [4,8,9,25].

B-complex vitamins are vital compounds for the proper functioning of the nervous system, the conversion of glucose into energy, and the metabolism of fats and proteins [26]. Buccal formulations of water soluble vitamins have been reported as patient-friendly dosage forms [27,28]. Factors like the saliva flow, chewing, swallowing, and speech may cause shearing in the oral cavity and obstruct the adhesion to the buccal mucosa, thus raising bioavailability issues; to address this, the burst release behavior of the formulations is able to simultaneously promote the diffusion of the API from the carrier and the rapid absorption by the mucus [29].

Thiamine hydrochloride (vitamin B1, THCl) is a hydrophilic vitamin, insoluble in the most common organic solvents, but soluble in polar solvents. It is vital for the maintenance of neuritis and polyneuritis. This vitamin is essential for the biosynthesis of gamma aminobutyric acid (GABA) and acetylcholine, the main neurotransmitter of the autonomic nervous system and also acts as a co-enzyme in the metabolism of fats, proteins and carbohydrates [30]. B1 deficiency may cause serious neurological and respiratory problems, dysfunction of the cardiovascular system, ophthalmoplegia, and nystagmus. Humans are unable to synthesize thiamine, and the recommended daily dose of B1 is 1.2 mg per day for adults and 0.2 mg per day for infants [31,32]. Grain and cereal-grain food products are the main source for B-complex vitamins, and it is claimed that the consumption of such products provides a positive effect in a variety of health-related conditions, e.g., diabetes, cardiovascular diseases, cancer of the colon, and lowering of blood cholesterol levels [33].

Nicotinic acid (vitamin B3, NA) is the precursor of nicotinamide adenine dinucleotide (NAD) and nicotinamide adenine dinucleotide phosphate (NADP), coenzymes that are involved in cellular redox reactions, along with nicotinamide. Moreover, NA seems to play a key role in the methylation of different substrates, including DNA, epinephrine and norepinephrine [34]. These findings suggest that NA is of highly importance for the human metabolic regulation and cell growth [35]. The NA vitamin is also involved in the metabolism of lipids, reducing the plasma levels of triglyceride and very low-density lipoprotein (VLDL), and resulting in raised levels of high-density lipoprotein (HDL). On top of that, B3 can cause blood vessel dilation, therefore it has a positive effect in various treatments such as hyperlipidemia, headache, vascular migraine, and cerebral arterial thrombosis. Some studies suggest that in can also manifest a neuroprotective behavior in stroke [36]. The recommended daily dose of NA is 15 mg per day for adults and it is synthesized from tryptophan, an amino acid inside the human body [37].

Both NA and THCl are B-group vitamins, essential for the nervous system and of high nutritional value. The buccal administration of these vitamins may have a very positive effect in cases of deficiency or poor nutrition. It has been reported that the combined NA and THCl administration provides anti-inflammatory properties and can reduce edema [38]. The aim of this study is to produce 2D-printed buccal films for the administration of these vitamins. Edible sheets were used as substrates for the deposition of the vitamin-loaded liquid ink and the formulation of the buccal films. These edible sugar sheets consist mainly of sugar monosaccharides and a polysaccharide, i.e., maltodextrin. These hydrophilic macromolecules contain hydrogen bond forming groups, thus favoring the adhesion to the buccal mucosa [39,40,41]. Propylene glycol was incorporated in the liquid ink as viscosity modifier, and has been reported to enhance the permeation and adhesion properties of buccal films [8,9,42]. Solubility studies, surface tension, and viscosity measurements were performed to determine the optimal liquid ink. Films with different doses can be produced by increasing the number of sequential passes from the printer, thus vitamin loading, and water uptake measurements are presented. Physiochemical characterization of the films, as well as in vitro release and in vitro permeation studies were also executed, to assess the developed formulations.

## 2. Results

### 2.1. Solubility Studies

The solubility studies for Nicotinic acid are presented in Table 1. The results indicated that NA is more soluble in water, as expected. The saturation solubility in water and in PG was 14.3 mg/mL and 8.4 mg/mL respectively. Regarding THCl, the solubility testing in water and in PG was terminated at a content of 198 mg/mL and 100 mg/mL, respectively, due to the observed increase in viscosity of the solutions, although, the solvents were capable of dissolving further amounts of the vitamin.

### 2.2. Viscosity and Surface Tension Measurements

Table 2 shows the viscosity values of 50:50, 60:40, 70:30, and 80:20 (% *v*/*v*) of H_2_O:PG binary mixtures. Vitamin-free binary mixtures were initially tested, whereas the addition of vitamins into the ink increased the viscosity. By increasing the concentration of both vitamins with a steady ratio between them, produced a very viscous ink solution, which was incapable of jetting through the ink cartridge. Finally, a vitamin content of 200 mg/mL and 12 mg/mL for THCL and NA, respectively, in a H_2_O:PG ratio of 60:40 (*v*/*v*) was selected as the optimal composition, following the evaluation of the 2D printing process.

### 2.3. Vitamin Loading and Water Uptake of the Developed Films

**Figure 1:**
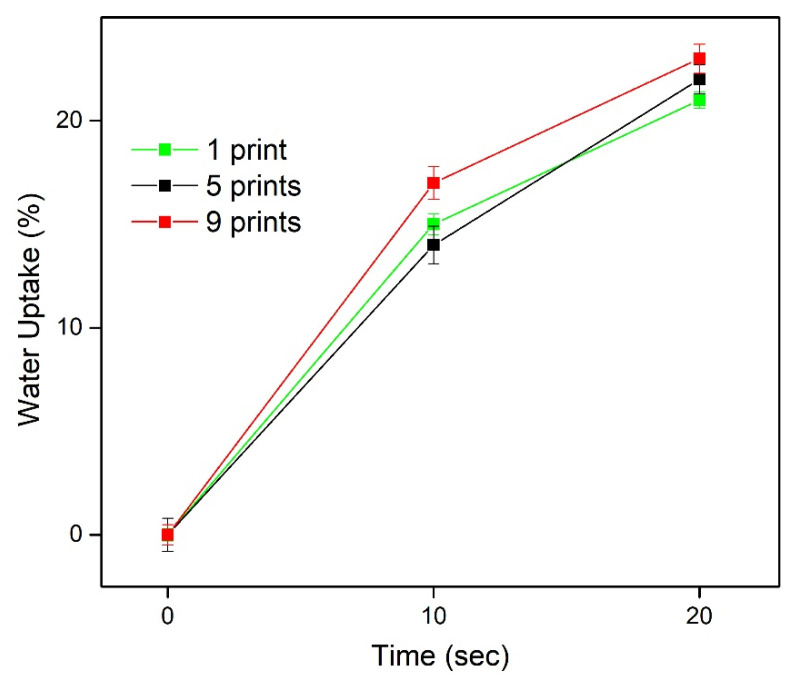
Water uptake of 1, 5, and 9 times printed films.

The amount of both vitamins in the formulated films was effectively increased, depending on the number of printing passes. Multiple passes through the printer produced three different formulations, with various doses of THCl and NA. The films were isolated manually from the substrate using a surgical blade; hence the content of vitamins is presented as mass of vitamin per mass of film. In detail, THCl content was 3.34 ± 0.03, 7.55 ± 0.09, and 9.39 ± 0.02 μg/mg film and NA content was 0.17 ± 0.001, 0.39 ± 0.004, and 0.57 ± 0.001 μg/mg film for 1-, 5-, and 9-times printed formulations, respectively. The water uptake results are presented in Figure 1. The water absorption capacity for all formulations was in the range 13–17% at 10 s and 19–23% at 20 s. Further immersion of the films in the medium promoted the extended deformation of structural integrity.

### 2.4. Thickness and Moisture Content

**Figure 2:**
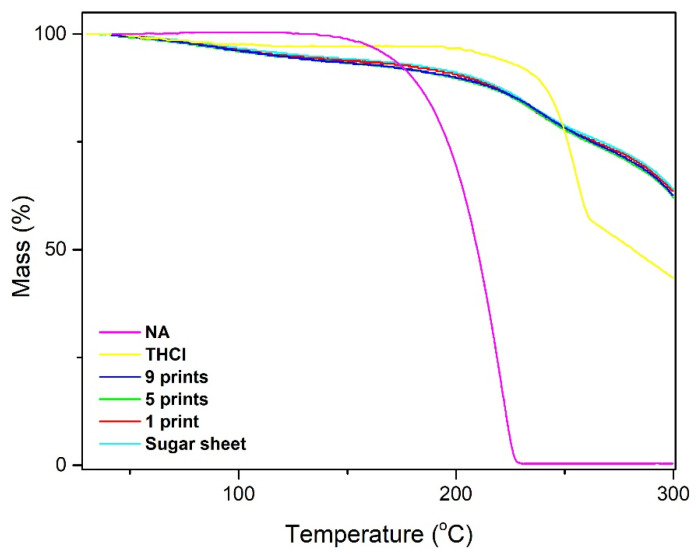
Thermo-gravimetric analysis of raw materials and printed formulations for the calculation of the moisture content.

The thickness of the plain substrate was 0.558 ± 0.001 mm, while the thickness of the 1-, 5-, and 9-printed specimens was 0.557 ± 0.005, 0.558 ± 0.007, and 0.558 ± 0.005 mm, respectively, indicating the absence of post-printing alterations on the geometric characteristics of the films (*p* > 0.05). The moisture content of the printed formulations and the plain sugar sheet was calculated by weighing the films before and after drying at 105 °C. The elimination of additional decomposition phenomena at this temperature range was based on the thermo-gravimetric analysis (TGA) evidence (Figure 2). The TGA thermograms revealed that the percentage of water is approximately 7% for all samples, indicated by mass losses at the temperature range of 50–150 °C, whereas thermal decomposition phenomena of THCl and NA appear to initiate at 210 °C and 160 °C, respectively. The moisture content calculated with the drying method was 8.21 ± 0.05%, 8.23 ± 0.07%, 8.25 ± 0.09%, and 8.29 ± 0.08% for sugar sheet, 1-, 5-, and 9-printed films, respectively (*p* > 0.05).

### 2.5. Physiochemical Characterization 

**Figure 3:**
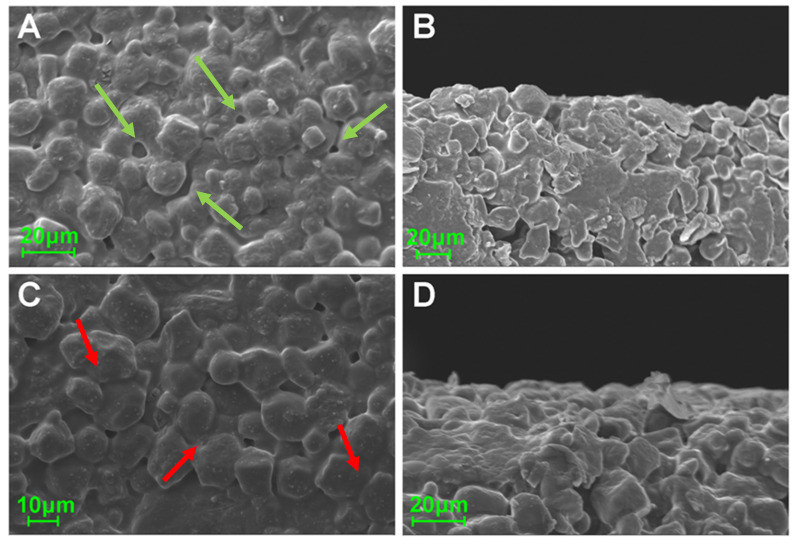
SEM micrographs of the upper and side surfaces of plain (**A**,**B**) and nine-pass (9)-printed (**C**,**D**) sugar sheets. The pores are marked with green arrows while the merged grains are marked with red arrows.

The SEM micrographs are presented in Figure 3. Upper and side views of the formulations revealed the rough surface and the presence of a pore network in the plain substrate matrix. Similar characteristics were apparent in 9-times printed films; however, the surface appears to be smoother and the substrate grains are merged in contrast to the plain substrate.

**Figure 4:**
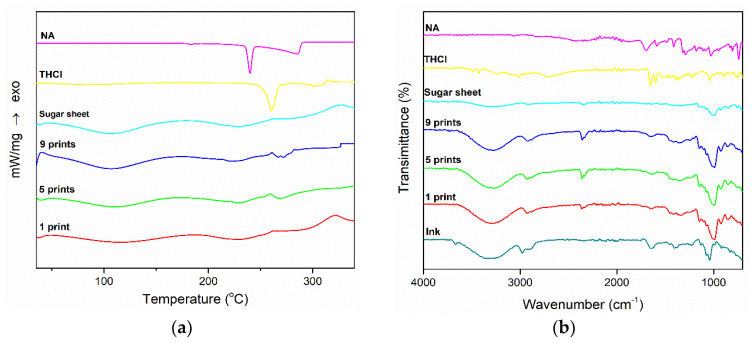
(**a**) DSC thermograms and (**b**) FTIR spectra of the samples.

The physiochemical properties of the films and the raw materials are shown in Figure 4. The DSC thermograms of THCl and NA presented a sharp endothermic peak at 260 °C and 240 °C, respectively. No such endotherms were recorded in the printed formulations. The formulations and the plain sugar sheet presented two broad endothermic peaks in the 70–150 °C and 190–250 °C range. Figure 4b illustrates the FTIR spectra of plain materials and printed formulations. In the spectrum of THCl, characteristic peaks at 1650, 1600, 1347, and 1438 cm^−1^ are apparent, whereas in the NA spectrum vibrations occurred in the range of 1770–1580 cm^−1^ and 1500–1300 cm^−1^. The plain sugar sheet, as well as the 1-, 5-, and 9-times printed films presented distinct vibrations in the area 3000–3500 cm^−1^ and 1200–700 cm^−1^. The characteristic vibrations of both vitamins were not detected in the developed formulations.

### 2.6. FTIR Data Analysis

#### 2.6.1. Two-Dimensional Fourier Transformation Infrared Correlation Spectroscopy (2D-COS-FTIR)

**Figure 5:**
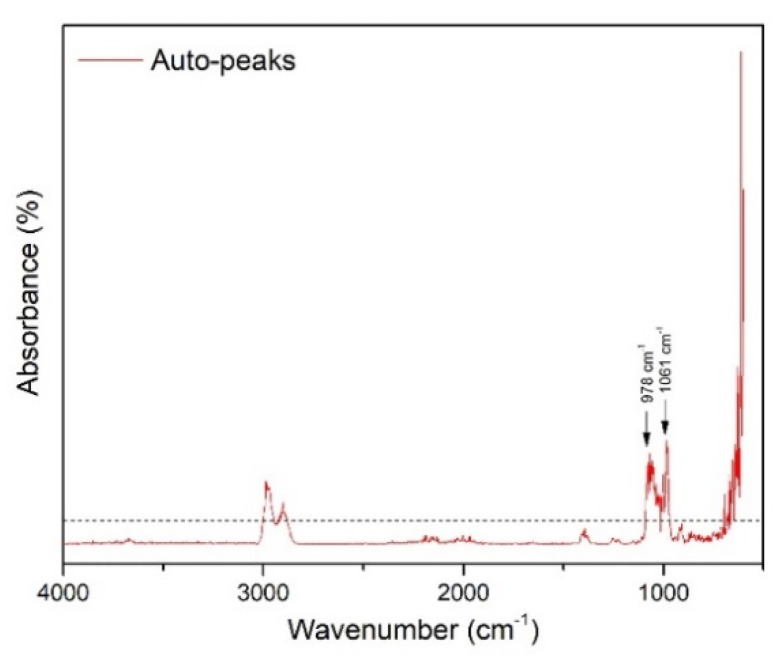
Two-dimensional Fourier transformation infrared correlation spectroscopy (2D-COS-FTIR) technique generated the auto-peaks plot.

2D-COS-FTIR was applied to monitor the dynamic spectral changes of the specimens during subsequent passes through the printer. The auto-peaks plot (Figure 5) was generated, based on the synchronous 2D-COS-FTIR analysis. The correlated data show that there are dynamic changes of the spectra, occurring around 1061 and 978 cm^−1^. These bands are attributed to the sugar sheet, indicating that the printing process alters the surface of the substrate. Considering the absence of further changes, it was suggested that the surface of the film is wetted by the ink as of the first printing pass, without any accumulation of ink, and the ink was instantly absorbed into the bulk of the substrate (film).

#### 2.6.2. Moving-Window 2D (MW2D) Correlation Spectroscopy

**Figure 6:**
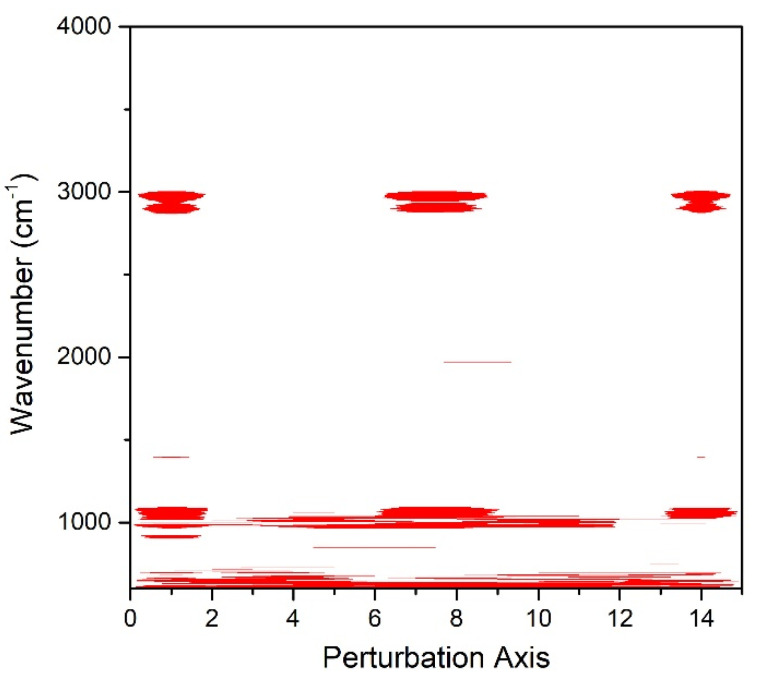
MW2D contour map for the visualization of the dynamic spectra changes.

MW2D analysis was applied and the contour map (Figure 6) was generated, to visualize the peak dynamic changes across the perturbation axis (sequential inkjet passes). The bands around 1061 cm^−1^ and 978 cm^−1^ exhibit changes over the investigated range of printing passes. Variations regarding the OH bonds of the sugar sheet were not detected, indicating the absence of ink accumulation onto the surface of the substrate.

### 2.7. In Vitro Release

**Figure 7:**
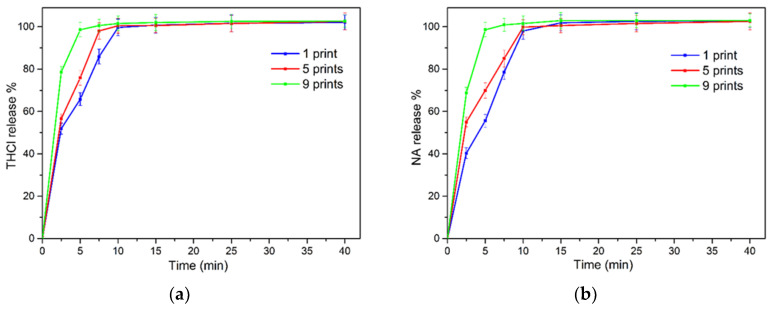
(**a**) Release profile of thiamine hydrochloride (THCl) and (**b**) NA.

The release of the vitamins in simulated saliva (SS) was monitored for 1 h. Figure 7 demonstrates the release profiles of the two compounds. All formulations released most of the loaded content within 10 min. In detail, 85%, 98%, and 100% of THCl and 78%, 85%, and 100% of NA was released at 7.5 min from 1, 5, and 9-times printed films, respectively. The obtained parameters from the curve fitting process are presented in Table 3. The data revealed that the profiles of all formulation for both THCL and NA release were optimally fitted to the first order kinetic model, with R^2^ values in the range 0.975–0.996.

### 2.8. In Vitro Permeation

**Figure 8:**
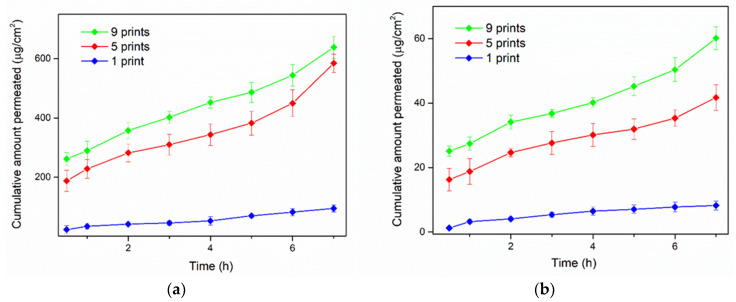
Cumulative transport of (**a**) THCl and (**b**) NA across the cellulose membrane.

Figure 8 illustrates the in vitro permeation profiles of the two vitamins, whereas Table 4 and Table 5 correspond to the permeation parameters of the study. It was evidenced that the increasing amount of vitamins content resulted in higher cumulative amounts that permeated across the cellulosic membrane. Accordingly, the steady-state flux and the apparent permeability coefficient were altered, in association with the number of printing passes. For THCl, the J_ss_ and P_app_ values were increased for 5 and 9-times printed films. Similarly, NA exhibited increased J_ss_ and P_app_ values of the 5- and 9- printed films, compared to the 1-printed specimen.

## 3. Discussion

THCl and NA are vitamins with low bioavailability when administered per os. THCl is absorbed in the jejunum and ileum by both passive and active uptake and reaches the systemic circulation after passing through the liver. Excess amounts of THCl are excreted through the renal clearance and the bioavailability is between 3.7% and 5.3% [43,44]. NA has a relative bioavailability of up to 25%, whereas 15–30% of the absorbed vitamin bounds to plasma proteins. NA has an extensive hepatic metabolism, which is also associated with the hepatic toxicity caused by higher doses of NA [45,46,47]. Buccal delivery of APIs has the main advantage of bypassing the first pass effect, including the avoidance of presystemic elimination within the GI tract. Thus, higher plasma concentrations of the drugs are achievable and the drug content within the buccal formulations is considerably lower, potentially reducing toxicity and other side effects [48]. To this context, we proposed a proof-of-concept approach for the buccal administration of these vitamins, whereas further optimization of these drug delivery systems is required.

Viscosity and surface tension are important factors to be considered in the development of liquid inks, intended for inkjet printing. Certain limits of the values of these parameters have been reported, regarding the available equipment; 1–30 mPa × sec for viscosity and 25–50 mN/m for surface tension [11,14]. Considering that the addition of vitamins in the ink will increase the viscosity, the composition of the plain H_2_O:PG binary mixture that was selected for further studies presented the optimal printing performance. In the case of 60:40 (% *v*/*v*) ratio, a dynamic viscosity of 4.476 mPa × s was calculated and an increasing amount of the two substances (under a steady ratio) was added in the mixture to produce a favorable ink solution. The optimal ink was selected with regard to the inkjet printing performance, i.e., the homogeneous distribution of the ink onto the edible substrate. Higher concentrations of the vitamins resulted in high-viscosity mixtures, unable to be jetted from the printer cartridge. Subsequently, the optimal ink was infused in the black ink cartridge. The buccal films were produced with sequential deposition of the vitamins onto the edible sugar sheets. By increasing the number of passes of the sugar sheet through the printer, various doses of the vitamins were produced. However, the 5- and 9-printed films presented lower vitamin dosing than expected, when compared to the 1-printed film. The disproportionate increase in dosing of inkjet-printed formulations, in relation to the number of printing passes, has been previously reported and associated with the shear forces that develop during the feeding process of the printer [49,50]. The conveyance of the edible sheets to the printing zone occurs upon traction of the substrate by the drive rollers of the device. Thus, at multiple printing passes, the generated shear forces often result in spreading of the ink beyond the printing boundaries of the predesigned patterns.

The morphological assessment revealed the rough surface of the sugar sheet and a smoothing of the surface of the ink-loaded film, due to partial solubilization of the superficial molecules. However, the printing process did not affect the overall geometry of the fabricated films, as revealed by the measured thickness values of the specimens. The commercial sugar sheet used in the present study is flexible and is intended to be used as substrate for printing edible substances. It was assumed that the amount of deposited ink onto the films was not efficient for altering the mechanical properties of the formulations. Although an increasing number of sequential printing passes was performed, the determined moisture content was similar for all specimens. This was indicative of the tightly bound water molecules in the substrate’s matrix, whereas a major amount of water molecules from the ink was instantly evaporated upon formation of liquid droplets at the printer’s orifice and the dispersion of droplets onto the substrates [8]. Moreover, the TGA data assured that the observed mass alterations upon drying were exclusively attributed to water evaporation, as thermal decomposition phenomena of the incorporated materials were observed at higher temperatures, compared to the drying conditions (105 °C). The DSC thermograms demonstrated sharp endothermic peaks at 260 °C and 240 °C for THCl and NA respectively, indicating the melting points of the vitamins [30,51]. These endotherms were not detected in the printed formulations, due to the amorphous state of the vitamins in the films or to the content of vitamins being below the detection limit of the instrument. At 5- and 9-prints, an endothermic peak around 260 °C was present as a sequence of an exothermic peak. These peaks indicate the recrystallization of THCl upon heating, followed by the melting of the substance [52]. Two broad endotherm peaks were detected in the temperature range of 70–150 °C and 190–250 °C for the plain sugar sheet and the formulations. These peaks are attributed to the evaporation of water and to the endothermic patterns of maltodextrin and sugar monosaccharides [50]. In the FTIR spectrum of THCl, characteristic peaks at 1650 cm^−1^ and 1600 cm^−1^ corresponded to the stretching bonds of C = N and aromatic C = C respectively [53] and peaks at 1347 cm^−1^ and 1438 cm^−1^ corresponded to the stretching modes of CH group of the pyrimidine ring [54]. Regarding NA, the stretching vibrations of C-C and COOH occurred in the range 1770–1580 cm^−1^, and the vibrations at 1500–1300 cm^−1^ were associated with the C-C and H-O bonds. Distinct vibrations of the sugar sheet in the area 3000–3500 cm^−1^ and 1200–700 cm^−1^ were present in all formulations. These vibrations were associated with the presence of maltodextrin, which is incorporated in the composition of the sugar sheet [55]. The absence or lower transmittance of the characteristic vibrations in the developed formulations including the ink solution, suggests that the vitamins content was either below the detection limit of the device or the vitamins were molecularly dissolved in the substrate matrix [50,56].

The 2D-COS-FTIR method allowed the monitoring of the surface changes at sequential inkjet printing. The correlated data indicate that there are no intensity changes of the bands that are associated with the surface matrix at 3200–3500 cm^−1^. These bands are attributed to the OH bonds of the sugar sheet. Since these peaks are present in the FTIR spectra of the individual films, but their intensity is not changed, it is considered that the ink is not accumulated on the surface of the films, but it is absorbed into the substrate matrix. The dynamic changes that occurred around 1061 cm^−1^ and at 978 cm^−1^ were attributed to the increase of the concentration of glycosidic bonds of maltodextrin [57]. The MW2D contour plot showed changes that occur across the investigated number of sequential prints, suggesting alterations on the surface of the sugar sheet that increased the contact area between the ATR crystal and the specimen. A possible mechanism that explains this observation is the partial surface erosion due to substrate dissolution phenomena. The ink acts as a solvent for the substrate leading the sugar to dissolve and recrystallize during the sequential cycles of printing. This dissolution/recrystallization process results in the decrease of porosity, leading to an increase in the observed intensity of these peaks. This hypothesis was supported by the SEM analysis, where the substrate grains (porosity) seem to merge during printing.

The total content of both vitamins was released within 10 min. Fitting of the obtained data on two non-linear models revealed the kinetics of the release profiles. Optimal fitting was evidenced on first-order kinetic model for the investigated vitamins-loaded sugar-sheet substrates, with significantly higher R^2^ values, compared to the Korsmeyer–Peppas model. The first-order kinetic model exhibits the gradual reduction of the release rate over time, and has been reported to represent the release behavior of water soluble APIs from porous matrices [58]. Regarding the in vitro permeation studies, variations in the composition of the dosage forms resulted in a significant increase of the cumulative amounts of the vitamins that permeated the cellulosic membrane. Increased amounts of vitamins also showed an increasing value for J_ss_ and for P_app_ (*p* < 0.05). This was attributed to the development of concentration gradients between the donor and the acceptor chambers, relevant to the increase in loading of the vitamins [50].

## 4. Materials and Methods

### 4.1. Materials

Thiamine Hydrochloride and Nicotinic acid were purchased from Fagron Hellas (Trikala, Greece). Propylene glycol (>99.5%) (PG) was purchased from Sigma-Aldrich (Steinheim, Germany). Décor Paper Plus edible sugar sheets (A4 dimension) were purchased from Kopyform GmbH (Beindersheim, Germany). The carbohydrate compounds of the substrates are sucrose, maltodextrin and dextrose. Further information on the composition of this product can be found in the manufacturer’s website (www.kopyform.com). All other compounds used in the current study were of analytical grade.

### 4.2. High Performance Liquid Chromatography (HPLC)

The quantification of the vitamins was accomplished via an HPLC system, which consisted of two LC-20AD pumps, a SIL-10AD autosampler (injection volume 100 μL), and a UV–DAD detector, with a Shimadzu LC Solution data system (Shimadzu, Kyoto, Japan). The wavelength chosen for the detection of both vitamins was 205 nm. The stationary phase was a Spherisorb^®^ CN-RP column (25 cm, 4.6 mm) with 5 μm particle size. The temperature of the samples during the analysis was set at 20 °C while the column temperature was 25 °C. A mixture consisting of 20 mmol/L sodium dihydrogen phosphate solution adjusted to pH 3.0 with 85% H_3_PO_4_ (aq) and Acetonitrile was used as mobile phase. The separation of the two vitamins was performed in gradient mode. Two different gradient run conditions were programmed for the in vitro release and permeation study as described in Table 6 and Table 7. The retention times for NA and THCl in SS was 6.6 min and 13.4 min, respectively, whereas the retention times in PBS was 7 min and 11.7 min for the release and permeation studies, respectively. The HPLC analytical method was validated according to ICH [59] guidelines in terms of linearity, selectivity (blank and spiked sample), limit of detection (LOD), limit of quantitation (LOQ), precision, and accuracy (Table 8). Stock standard solutions of both vitamins were prepared in methanol and thereafter appropriate dilutions using SS pH 6.8 and PBS pH 7.4 as diluent were performed.

### 4.3. Solubility Studies

The solubility studies for each vitamin were conducted in distilled water as the main solvent, and in PG as surface tension and viscosity modifier. Airtight glass vials, containing 10 mL of each solvent, were placed under magnetic stirring (200 rpm). Pre-weighed amounts of THCl and NA were gradually added in the solvents, until a cloudy mixture was produced, suggesting that saturation solubility has been achieved. After 24 h, 5 mL of each mixture were sampled, centrifuged at 4000 rcf for 30 min, and filtered through 0.45 μm Whatman Nylon filters (Whatman International Ltd., Maidstone, UK). The supernatants were collected and the saturation solubility of THCl and NA in each solvent was quantified by HPLC.

### 4.4. Ink Development

The two solvents were mixed under different ratios, to produce a vitamin-loaded ink with appropriate rheological behavior. A Micro Ostwald viscometer (SI Analytics GmbH, Mainz, Germany) was used to determine the kinematic viscosity (ν) of three different vitamin-free solvent ratios, in triplicate. The dynamic viscosity (n) was calculated from the equation n = ν × *p*. The density (*p*) of each sample was determined gravimetrically. The addition of vitamins in the ink formulation can alter the rheological behavior of the ink samples, so additional rheological measurements were conducted after the vitamin loading. The surface tension of the optimal samples was determined by a CAM 200 contact angle goniometer (KSV Instruments, Helsinki, Finland), and the data analysis was performed with the aid of One Attension software (Biolin Scientific, Espoo, Finland).

### 4.5. Printing of Buccal Films

The buccal films were prepared using a Canon MG2950 (Canon Greece, Athens, Greece). The cartridge with the black ink was cleaned and filled with the vitamin solution. Word 2010 (Microsoft Inc., Redmond, DC, USA) was used to generate square patterns of 2 cm × 2 cm onto the sugar sheet substrate. In order to increase the amount of the vitamins onto the substrate, the printing process was performed with one, five or nine sequential passes. After the printing process, the films were isolated manually from the substrate by using a surgical blade.

### 4.6. Vitamins Loading and Water Uptake

The produced films were dispersed in 50 mL distilled water and kept in sealed glass vials for 4 h under magnetic stirring (300 rpm). 5 mL of each vial was withdrawn and centrifuged at 4000 rcf for 20 min. The supernatant was collected and analyzed by HPLC, to determine the amount of the vitamins on each film. To evaluate the water absorption capacity, printed films were immersed in Petri dishes, containing 1 mL of simulated saliva (SS) pH 6.8 (0.8% sodium chloride, 0.019% potassium phosphate-monobasic and 0.238% sodium phosphate-dibasic (*w*/*v*)) [60]. At regular time intervals, the films were removed from the Petri dishes, whipped out carefully to remove excess water and weighed. The water uptake (WU) was estimated as %WU = ((w2 − w1) × 100)/w1)), where w1 and w2 is the weight of each film before and after immersion in the SS medium.

### 4.7. Thickness and Moisture Content

The thickness of the three formulations and the plain sugar sheet was determined by a 0–25 mm (±0.01 mm) handheld caliper (Standard Gage, Hexagon Metrology, Stockholm, Sweden). Five random areas of each film were measured to obtain the average value (*n* = 3). The moisture content was calculated with the drying method. Sugar sheet, 1-, 5-, and 9-printed films were accurately weighed before and after drying at 105 °C for 4 h (*n* = 6). The mass loss observed after 4 h of drying was attributed to water evaporation of each film. To ensure the absence of simultaneous thermal decomposition phenomena at this temperature, thermo-gravimetric analysis (Shimadzu TGA-50 instrument, Tokyo, Japan) was performed. The samples (3 mg) were placed in aluminum pans and were heated in the 30–300 °C range, with a heating rate of 10 °C/min under nitrogen environment.

### 4.8. Physiochemical Characterization

The morphological characteristics of the printed films were evaluated by scanning electron microscopy (SEM), using a Zeiss SUPRA 35VP microscope (Zeiss, Oberkochen, Germany). Thermal analysis of the printed formulations was carried out by differential scanning calorimetry (DSC). The thermograms of the films and the raw materials were recorded by a DSC 204 F1 Phoenix (Netzsch, Selb, Germany) instrument. Samples, weighing approximately 5 mg, were placed in aluminum pans and were heated at a rate of 10 °C/min, between 30–330 °C. Fourier Transform Infrared Spectroscopy (FTIR) studies were also conducted by an IRPrestige-21 (Shimadzu, Kyoto, Japan) instrument. The spectrum of each sample was recorded in the range 650–4000 cm^−1^, with 2 cm^−1^ resolution.

### 4.9. FTIR Data Analysis

#### 4.9.1. Two-Dimensional Fourier Transformation Infrared Correlation Spectroscopy (2D-COS-FTIR)

2DCorrFTIR was applied to monitor the dynamic spectral changes of the films’ surface during subsequent passes through the printer device. Specimens printed 1 to 14 times were prepared and the data were analyzed using 2D Shige (2DShige©, Shigeaki Morita, Kwansei-Gakuin University, 2004–2005) [61,62].

#### 4.9.2. Moving-Window 2D Correlation (MW2D) Spectroscopy

MW2D technique was applied to monitor the induced spectral variations on the drug loaded specimens, during sequential passes through the printer. The spectral data were analyzed using 2D Shige package, through the generation of a 2D map spread of the spectral variables as a function of the external perturbation (1–14 printing passes) [63].

### 4.10. In Vitro Studies

#### 4.10.1. Thiamine Hydrochloride and Nicotinic Acid Release in Simulated Saliva

The in vitro release studies were conducted in SS, in triplicate. The printed formulations of different doses were enclosed in metal grids and submerged in double-walled glass vessels containing 50 mL of SS. The vessels containing the films were kept under gentle agitation (100 rpm) at 37 °C. 1 mL from each vessel was withdrawn at pre-determined time points, centrifuged at 4000 rcf for 20 min, filtered through a 0.45 μm filter and analyzed by HPLC. Two kinetic models were fitted on the release data (first order, Korsmeyer-Peppas), using the software SigmaPlot v.12.5 (Systat Software, Inc., Chicago, IL, USA) and a curve fit library (release.jfl).

#### 4.10.2. Permeation Studies

The in vitro permeation of the two vitamins across a cellulosic membrane (Dialysis Tubing, MW cut-off, Sigma-Aldrich, Steinheim, Germany) was studied in Franz vertical diffusion cells (diffusion area 4.9 cm^2^, compartment volume 20 mL). In order for the films to fit properly into the donor compartment, round films (r = 1.128 cm) with a surface area of approximately 4 cm^2^ were printed. The acceptor and the donor chambers were filled with degassed PBS pH 7.4 and SS pH 6.8, respectively, and maintained under constant magnetic stirring (110 rpm) at 37 °C, whereas the cellulose membrane was properly mounted between the two compartments. Samples of 1 mL were withdrawn from the acceptor compartment at predetermined time intervals, centrifuged at 4000 rcf for 20 min, filtered through a 0.45 m filter, and analyzed by HPLC. The amount of the vitamins permeated through the membrane was plotted against time, and the slope of the linear section of the curve determined the steady-state flux (J_ss_). The apparent permeability coefficient (P_app_) was calculated as P_app_ = J_ss_/C_d_, where C_d_ indicates the concentration of THCl and NA in the donor compartment.

### 4.11. Statistical Analysis

The data are presented as the mean ± SD. The significance level is set at *p* < 0.05 (Student’s *t*-test).

## 5. Conclusions

Buccal films of B-complex vitamins were developed via 2D printing technology, a new and safe way for producing drug or vitamin loaded formulations. The solubility studies revealed that the vitamins are very soluble in distilled water and PG, two non-toxic and low-cost solvents. The increased solubility suggests that the doses could be easily adjusted to specific needs, regarding the amount of vitamins dissolved in the ink formulation and the number of sequential printing passes. The physicochemical characterization of the printed films presented the morphology of the formulations and revealed the amorphous state of the vitamins. The release profiles indicated the rapid release from the matrix, whereas the permeation profiles showed that increasing the number of sequential passes improved the permeability of each vitamin.

## Figures and Tables

**Table 1:** Solubility studies for nicotinic acid (NA).

Solvent	Solubility (mg/mL)
Distilled Water	14.3
Propylene glycol	8.4

**Table 2:** Viscosity and surface tension measurements of the liquid inks.

H_2_O:PG Ratio (% *v*/*v*)	THCl (mg/mL)	NA (mg/mL)	Kinematic Viscosity (mm^2^/s)	Dynamic Viscosity (mPa × s)	Surface Tension (mN/m)
80:20	-	-	2.105 ± 0.02	2.157 ± 0.03	-
70:30	-	-	3.087 ± 0.06	3.173 ± 0.02	-
60:40	-	-	4.294 ± 0.04	4.476 ± 0.08	26.8 ± 0.05
60:40	200.0	12.0	5.356 ± 0.03	5.525 ± 0.01	28.4 ± 0.03
60:40	230.0	13.8	12.316 ± 0.07	12.598 ± 0.03	-

**Table 3:** Kinetic models fitting parameters.

Formulation	Vitamin	First Order		Korsmeyer–Peppas		
		k	R^2^	k	n	R^2^
1-print	THCl	0.29	0.986	53.04	0.20	0.929
	NA	0.21	0.975	43.14	0.27	0.898
5-prints	THCl	0.34	0.990	61.66	0.16	0.930
	NA	0.28	0.988	55.45	0.19	0.943
9-prints	THCl	0.64	0.996	83.32	0.07	0.978
	NA	0.52	0.990	77.35	0.09	0.950

**Table 4:** Permeation parameters for THCl.

Printed Layers	J_ss_ (μg × cm^−2^ × h^−1^)	P_app_∙10^−4^(cm × h^−1^)
1	0.206 ± 0.06	1.322 ± 0.39
5	1.684 ± 0.32	4.700 ± 0.40
9	1.710 ± 0.41	6.690 ± 1.80

**Table 5:** Permeation parameters for NA.

Printed Layers	J_ss_ (μg × cm^−2^ × h^−1^)	P_app_∙10^−4^(cm × h^−1^)
1	0.009 ± 0.001	1.204 ± 0.150
5	0.082 ± 0.018	4.085 ± 0.910
9	0.142 ± 0.050	11.188 ± 1.120

**Table 6:** Gradient conditions for the elution of THCl and NA in simulated saliva (SS).

Time (min)	ACN (%)	Phosphate Buffer (%)	Flow Rate (mL/min)
0–6	2	98	0.5
6–8	5	95	0.5
8–13	15	85	1.0
13–15	25	75	1.0
15–18	2	98	0.5

**Table 7:** Gradient conditions for the elution of THCl and NA in PBS.

Time (min)	ACN (%)	Phosphate Buffer (%)	Flow Rate (mL/min)
0–6	2	98	0.5
6–7	5	95	0.5
7–11	10	90	0.5
11–15	2	98	0.5

**Table 8:** Data analysis of validation tests.

		Release Tests	Permeation Tests
	Concentration range	0.596–19.2 μg/mL	0.632–24.96 μg/ml
	Calibration curve	y = 785579.7x − 257656	y = 604067.2x − 206475.1
	R^2^	0.9999	0.9999
NA	Limit of detection (LOD)	0.196	0.086
	Limit of quantitation (LOQ)	0.595	0.261
	Mean %Recovery	>95.6%	>96.1
	Mean %RSD	3.1	2.01
	Concentration range	1.184–20.16 μg/ml	1.232–184.8 μg/ml
	Calibration curve	282969.4x − 6572	260781.98x − 49662
THCl	R^2^	0.9992	0.9996
	LOD	0.336	0.591
	LOQ	1.017	1.789
	Mean %Recovery	>97.3	>98.4
	Mean %RSD average	3.7	2.51
